# Supplementary material for: Particularities of the changes in young swimmers’ body adaptation to the stimuli of physical and mental stress in sports training process
Source: PeerJ. 2021 Jun 23;9:e11659. doi: 10.7717/peerj.11659 (PMC8234917; doi:10.7717/peerj.11659)
Supplement: Supplemental Information 2 [file peerj-09-11659-s002.rtf]

Cohen-Williamson Test

RELIABILITY
  /VARIABLES=VAR00001 VAR00002 VAR00003 VAR00004 VAR00006 VAR00007 VAR00008 VAR00009 VAR00010
    VAR00011 VAR00012 VAR00013 VAR00014
  /SCALE('ALL VARIABLES') ALL
  /MODEL=ALPHA
  /SUMMARY=TOTAL.


Reliability


Notes	
Output Created	04-MAR-2021 04:18:52	
Comments		
Input	Active Dataset	DataSet0	
	Filter	<none>	
	Weight	<none>	
	Split File	<none>	
	N of Rows in Working Data File	6	
	Matrix Input		
Missing Value Handling	Definition of Missing	User-defined missing values are treated as missing.	
	Cases Used	Statistics are based on all cases with valid data for all variables in the procedure.	
Syntax	RELIABILITY
  /VARIABLES=VAR00001 VAR00002 VAR00003 VAR00004 VAR00006 VAR00007 VAR00008 VAR00009 VAR00010
    VAR00011 VAR00012 VAR00013 VAR00014
  /SCALE('ALL VARIABLES') ALL
  /MODEL=ALPHA
  /SUMMARY=TOTAL.	
Resources	Processor Time	00:00:00.02	
	Elapsed Time	00:00:00.09	


[DataSet0] 


Scale: ALL VARIABLES


Case Processing Summary	
	N	%	
Cases	Valid	6	100.0	
	Excludeda	0	.0	
	Total	6	100.0	

a. Listwise deletion based on all variables in the procedure.	


Reliability Statistics	
Cronbach's Alpha	N of Items	
.545	13	


Item-Total Statistics	
	Scale Mean if Item Deleted	Scale Variance if Item Deleted	Corrected Item-Total Correlation	Cronbach's Alpha if Item Deleted	
VAR00001	24.6667	16.267	.543	.478	
VAR00002	24.3333	18.267	.153	.537	
VAR00003	23.8333	21.367	-.559	.623	
VAR00004	24.5000	18.300	.000	.568	
VAR00006	24.3333	18.667	-.041	.573	
VAR00007	24.5000	19.900	-.220	.610	
VAR00008	24.1667	14.167	.924	.395	
VAR00009	24.1667	22.967	-.560	.681	
VAR00010	24.3333	18.267	.021	.561	
VAR00011	23.6667	10.667	.711	.320	
VAR00012	22.6667	12.667	.551	.408	
VAR00013	24.3333	13.467	.628	.405	
VAR00014	24.5000	13.100	.880	.361	
RELIABILITY
  /VARIABLES=VAR00001 VAR00002 VAR00003 VAR00004 VAR00006 VAR00007 VAR00008 VAR00009 VAR00010
    VAR00011 VAR00012 VAR00013 VAR00014
  /SCALE('ALL VARIABLES') ALL
  /MODEL=ALPHA
  /SUMMARY=TOTAL.


Reliability


Notes	
Output Created	04-MAR-2021 04:24:28	
Comments		
Input	Active Dataset	DataSet0	
	Filter	<none>	
	Weight	<none>	
	Split File	<none>	
	N of Rows in Working Data File	6	
	Matrix Input		
Missing Value Handling	Definition of Missing	User-defined missing values are treated as missing.	
	Cases Used	Statistics are based on all cases with valid data for all variables in the procedure.	
Syntax	RELIABILITY
  /VARIABLES=VAR00001 VAR00002 VAR00003 VAR00004 VAR00006 VAR00007 VAR00008 VAR00009 VAR00010
    VAR00011 VAR00012 VAR00013 VAR00014
  /SCALE('ALL VARIABLES') ALL
  /MODEL=ALPHA
  /SUMMARY=TOTAL.	
Resources	Processor Time	00:00:00.00	
	Elapsed Time	00:00:00.00	


Warnings	
Scale has zero variance items.	


Scale: ALL VARIABLES


Case Processing Summary	
	N	%	
Cases	Valid	6	100.0	
	Excludeda	0	.0	
	Total	6	100.0	

a. Listwise deletion based on all variables in the procedure.	

Reliability Statistics	
Cronbach's Alpha	N of Items	
.602	13	


Item-Total Statistics	
	Scale Mean if Item Deleted	Scale Variance if Item Deleted	Corrected Item-Total Correlation	Cronbach's Alpha if Item Deleted	
VAR00001	26.1667	8.967	.927	.406	
VAR00002	25.5000	11.500	.685	.519	
VAR00003	25.3333	11.467	.353	.558	
VAR00004	25.8333	10.167	.893	.458	
VAR00006	26.0000	12.400	.556	.551	
VAR00007	25.1667	17.367	-.805	.712	
VAR00008	25.3333	18.267	-.671	.756	
VAR00009	26.6667	11.867	.758	.527	
VAR00010	26.6667	13.867	.044	.608	
VAR00011	25.1667	9.367	.591	.481	
VAR00012	24.1667	9.367	.591	.481	
VAR00013	26.1667	13.367	.022	.631	
VAR00014	25.8333	14.167	.000	.606	

RELIABILITY
  /VARIABLES=VAR00001 VAR00002 VAR00003 VAR00004 VAR00006 VAR00007 VAR00008 VAR00009 VAR00010
    VAR00011 VAR00012 VAR00013 VAR00014
  /SCALE('ALL VARIABLES') ALL
  /MODEL=ALPHA
  /SUMMARY=TOTAL.


Reliability


Notes	
Output Created	04-MAR-2021 04:25:51	
Comments		
Input	Active Dataset	DataSet0	
	Filter	<none>	
	Weight	<none>	
	Split File	<none>	
	N of Rows in Working Data File	6	
	Matrix Input		
Missing Value Handling	Definition of Missing	User-defined missing values are treated as missing.	
	Cases Used	Statistics are based on all cases with valid data for all variables in the procedure.	
Syntax	RELIABILITY
  /VARIABLES=VAR00001 VAR00002 VAR00003 VAR00004 VAR00006 VAR00007 VAR00008 VAR00009 VAR00010
    VAR00011 VAR00012 VAR00013 VAR00014
  /SCALE('ALL VARIABLES') ALL
  /MODEL=ALPHA
  /SUMMARY=TOTAL.	
Resources	Processor Time	00:00:00.00	
	Elapsed Time	00:00:00.00	


Scale: ALL VARIABLES


Case Processing Summary	
	N	%	
Cases	Valid	6	100.0	
	Excludeda	0	.0	
	Total	6	100.0	

a. Listwise deletion based on all variables in the procedure.	


Reliability Statistics	
Cronbach's Alpha	N of Items	
.588	13	


Item-Total Statistics	
	Scale Mean if Item Deleted	Scale Variance if Item Deleted	Corrected Item-Total Correlation	Cronbach's Alpha if Item Deleted	
VAR00001	28.0000	8.800	.829	.397	
VAR00002	27.5000	10.300	.967	.445	
VAR00003	27.3333	11.867	.525	.527	
VAR00004	27.3333	9.467	.770	.430	
VAR00006	27.8333	15.767	-.596	.660	
VAR00007	27.8333	15.767	-.596	.660	
VAR00008	27.8333	14.967	-.359	.637	
VAR00009	28.5000	13.100	.151	.583	
VAR00010	28.6667	16.667	-.696	.689	
VAR00011	26.6667	9.067	.622	.449	
VAR00012	26.5000	7.900	.848	.359	
VAR00013	28.1667	12.567	.438	.550	
VAR00014	27.8333	14.167	-.153	.673	


RELIABILITY
  /VARIABLES=VAR00001 VAR00002 VAR00003 VAR00004 VAR00006 VAR00007 VAR00008 VAR00009 VAR00010
    VAR00011 VAR00012 VAR00013 VAR00014
  /SCALE('ALL VARIABLES') ALL
  /MODEL=ALPHA
  /SUMMARY=TOTAL.


Reliability


Notes	
Output Created	04-MAR-2021 04:26:35	
Comments		
Input	Active Dataset	DataSet0	
	Filter	<none>	
	Weight	<none>	
	Split File	<none>	
	N of Rows in Working Data File	6	
	Matrix Input		
Missing Value Handling	Definition of Missing	User-defined missing values are treated as missing.	
	Cases Used	Statistics are based on all cases with valid data for all variables in the procedure.	
Syntax	RELIABILITY
  /VARIABLES=VAR00001 VAR00002 VAR00003 VAR00004 VAR00006 VAR00007 VAR00008 VAR00009 VAR00010
    VAR00011 VAR00012 VAR00013 VAR00014
  /SCALE('ALL VARIABLES') ALL
  /MODEL=ALPHA
  /SUMMARY=TOTAL.	
Resources	Processor Time	00:00:00.02	
	Elapsed Time	00:00:00.02	


Scale: ALL VARIABLES


Case Processing Summary	
	N	%	
Cases	Valid	6	100.0	
	Excludeda	0	.0	
	Total	6	100.0	

a. Listwise deletion based on all variables in the procedure.	


Reliability Statistics	
Cronbach's Alpha	N of Items	
.423	13	


Item-Total Statistics	
	Scale Mean if Item Deleted	Scale Variance if Item Deleted	Corrected Item-Total Correlation	Cronbach's Alpha if Item Deleted	
VAR00001	30.3333	15.067	.716	.212	
VAR00002	30.3333	20.267	.030	.438	
VAR00003	29.8333	13.767	.870	.137	
VAR00004	29.8333	22.167	-.187	.518	
VAR00006	29.6667	24.667	-.374	.590	
VAR00007	30.1667	20.567	.036	.431	
VAR00008	30.0000	17.200	.519	.304	
VAR00009	30.3333	22.667	-.242	.488	
VAR00010	30.0000	18.800	.505	.348	
VAR00011	30.0000	18.800	.276	.371	
VAR00012	29.3333	21.467	-.184	.576	
VAR00013	30.0000	19.200	.417	.364	
VAR00014	30.1667	14.967	.929	.185	


Cohen Perceived Stress Test

RELIABILITY
  /VARIABLES=VAR00001 VAR00002 VAR00003 VAR00004 VAR00005 VAR00006 VAR00007 VAR00008 VAR00009
    VAR00010
  /SCALE('ALL VARIABLES') ALL
  /MODEL=ALPHA
  /SUMMARY=TOTAL.


Reliability


Notes	
Output Created	04-MAR-2021 05:07:16	
Comments		
Input	Active Dataset	DataSet0	
	Filter	<none>	
	Weight	<none>	
	Split File	<none>	
	N of Rows in Working Data File	6	
	Matrix Input		
Missing Value Handling	Definition of Missing	User-defined missing values are treated as missing.	
	Cases Used	Statistics are based on all cases with valid data for all variables in the procedure.	
Syntax	RELIABILITY
  /VARIABLES=VAR00001 VAR00002 VAR00003 VAR00004 VAR00005 VAR00006 VAR00007 VAR00008 VAR00009
    VAR00010
  /SCALE('ALL VARIABLES') ALL
  /MODEL=ALPHA
  /SUMMARY=TOTAL.	
Resources	Processor Time	00:00:00.00	
	Elapsed Time	00:00:00.00	


[DataSet0] 


Scale: ALL VARIABLES


Case Processing Summary	
	N	%	
Cases	Valid	6	100.0	
	Excludeda	0	.0	
	Total	6	100.0	

a. Listwise deletion based on all variables in the procedure.	


Reliability Statistics	
Cronbach's Alpha	N of Items	
.273	10	


Item-Total Statistics	
	Scale Mean if Item Deleted	Scale Variance if Item Deleted	Corrected Item-Total Correlation	Cronbach's Alpha if Item Deleted	
VAR00001	16.3333	5.067	.562	.030	
VAR00002	16.3333	7.467	-.231	.382	
VAR00003	15.8333	6.967	-.181	.441	
VAR00004	14.5000	6.700	.095	.257	
VAR00005	14.6667	7.067	-.194	.446	
VAR00006	16.3333	5.067	.562	.030	
VAR00007	14.5000	5.900	.164	.216	
VAR00008	14.5000	6.700	.095	.257	
VAR00009	16.3333	7.067	-.119	.340	
VAR00010	16.6667	3.867	.789	-.233a	

a. The value is negative due to a negative average covariance among items. This violates reliability model assumptions. You may want to check item codings.	

RELIABILITY
  /VARIABLES=VAR00001 VAR00002 VAR00003 VAR00004 VAR00005 VAR00006 VAR00007 VAR00008 VAR00009
    VAR00010
  /SCALE('ALL VARIABLES') ALL
  /MODEL=ALPHA
  /SUMMARY=TOTAL.


Reliability


Notes	
Output Created	04-MAR-2021 05:08:20	
Comments		
Input	Active Dataset	DataSet0	
	Filter	<none>	
	Weight	<none>	
	Split File	<none>	
	N of Rows in Working Data File	6	
	Matrix Input		
Missing Value Handling	Definition of Missing	User-defined missing values are treated as missing.	
	Cases Used	Statistics are based on all cases with valid data for all variables in the procedure.	
Syntax	RELIABILITY
  /VARIABLES=VAR00001 VAR00002 VAR00003 VAR00004 VAR00005 VAR00006 VAR00007 VAR00008 VAR00009
    VAR00010
  /SCALE('ALL VARIABLES') ALL
  /MODEL=ALPHA
  /SUMMARY=TOTAL.	
Resources	Processor Time	00:00:00.00	
	Elapsed Time	00:00:00.00	


Scale: ALL VARIABLES


Case Processing Summary	
	N	%	
Cases	Valid	6	100.0	
	Excludeda	0	.0	
	Total	6	100.0	

a. Listwise deletion based on all variables in the procedure.	


Reliability Statistics	
Cronbach's Alpha	N of Items	
.097	10	


Item-Total Statistics	
	Scale Mean if Item Deleted	Scale Variance if Item Deleted	Corrected Item-Total Correlation	Cronbach's Alpha if Item Deleted	
VAR00001	19.8333	4.567	.038	.082	
VAR00002	19.5000	3.500	.207	-.107a	
VAR00003	18.6667	5.867	-.302	.243	
VAR00004	18.1667	6.967	-.599	.398	
VAR00005	18.5000	5.500	-.209	.259	
VAR00006	19.5000	3.500	.828	-.364a	
VAR00007	18.6667	5.467	-.156	.178	
VAR00008	18.6667	5.467	-.156	.178	
VAR00009	19.5000	3.500	.828	-.364a	
VAR00010	19.5000	3.500	.393	-.236a	

a. The value is negative due to a negative average covariance among items. This violates reliability model assumptions. You may want to check item codings.	


RELIABILITY
  /VARIABLES=VAR00001 VAR00002 VAR00003 VAR00004 VAR00005 VAR00006 VAR00007 VAR00008 VAR00009
    VAR00010
  /SCALE('ALL VARIABLES') ALL
  /MODEL=ALPHA
  /SUMMARY=TOTAL.


Reliability


Notes	
Output Created	04-MAR-2021 05:09:03	
Comments		
Input	Active Dataset	DataSet0	
	Filter	<none>	
	Weight	<none>	
	Split File	<none>	
	N of Rows in Working Data File	6	
	Matrix Input		
Missing Value Handling	Definition of Missing	User-defined missing values are treated as missing.	
	Cases Used	Statistics are based on all cases with valid data for all variables in the procedure.	
Syntax	RELIABILITY
  /VARIABLES=VAR00001 VAR00002 VAR00003 VAR00004 VAR00005 VAR00006 VAR00007 VAR00008 VAR00009
    VAR00010
  /SCALE('ALL VARIABLES') ALL
  /MODEL=ALPHA
  /SUMMARY=TOTAL.	
Resources	Processor Time	00:00:00.00	
	Elapsed Time	00:00:00.01	


Scale: ALL VARIABLES


Case Processing Summary	
	N	%	
Cases	Valid	6	100.0	
	Excludeda	0	.0	
	Total	6	100.0	

a. Listwise deletion based on all variables in the procedure.	

Reliability Statistics	
Cronbach's Alphaa	N of Items	
-2.798	10	

a. The value is negative due to a negative average covariance among items. This violates reliability model assumptions. You may want to check item codings.	

Item-Total Statistics	
	Scale Mean if Item Deleted	Scale Variance if Item Deleted	Corrected Item-Total Correlation	Cronbach's Alpha if Item Deleted	
VAR00001	21.0000	1.600	.577	-5.344a	
VAR00002	20.5000	1.900	-.229	-3.553a	
VAR00003	20.0000	1.600	.189	-5.063a	
VAR00004	19.6667	4.667	-.779	-1.029a	
VAR00005	19.8333	5.367	-.881	-.727a	
VAR00006	20.6667	2.267	.217	-3.507a	
VAR00007	19.5000	6.700	-.917	-.269a	
VAR00008	19.8333	4.967	-.806	-.876a	
VAR00009	20.8333	.567	-.217	-12.441a	
VAR00010	20.6667	1.467	.112	-5.420a	

a. The value is negative due to a negative average covariance among items. This violates reliability model assumptions. You may want to check item codings.	

RELIABILITY
  /VARIABLES=VAR00001 VAR00002 VAR00003 VAR00004 VAR00005 VAR00006 VAR00007 VAR00008 VAR00009
    VAR00010
  /SCALE('ALL VARIABLES') ALL
  /MODEL=ALPHA
  /SUMMARY=TOTAL.


Reliability


Notes	
Output Created	04-MAR-2021 05:09:48	
Comments		
Input	Active Dataset	DataSet0	
	Filter	<none>	
	Weight	<none>	
	Split File	<none>	
	N of Rows in Working Data File	6	
	Matrix Input		
Missing Value Handling	Definition of Missing	User-defined missing values are treated as missing.	
	Cases Used	Statistics are based on all cases with valid data for all variables in the procedure.	
Syntax	RELIABILITY
  /VARIABLES=VAR00001 VAR00002 VAR00003 VAR00004 VAR00005 VAR00006 VAR00007 VAR00008 VAR00009
    VAR00010
  /SCALE('ALL VARIABLES') ALL
  /MODEL=ALPHA
  /SUMMARY=TOTAL.	
Resources	Processor Time	00:00:00.02	
	Elapsed Time	00:00:00.00	

Scale: ALL VARIABLES

Case Processing Summary	
	N	%	
Cases	Valid	6	100.0	
	Excludeda	0	.0	
	Total	6	100.0	

a. Listwise deletion based on all variables in the procedure.	


Reliability Statistics	
Cronbach's Alphaa	N of Items	
-1.401	10	

a. The value is negative due to a negative average covariance among items. This violates reliability model assumptions. You may want to check item codings.	

Item-Total Statistics	
	Scale Mean if Item Deleted	Scale Variance if Item Deleted	Corrected Item-Total Correlation	Cronbach's Alpha if Item Deleted	
VAR00001	21.8333	2.567	-.075	-1.607a	
VAR00002	21.6667	1.867	.047	-2.411a	
VAR00003	21.3333	1.467	.369	-3.580a	
VAR00004	20.5000	2.700	-.081	-1.528a	
VAR00005	20.3333	4.267	-.612	-.598a	
VAR00006	21.5000	3.500	-.393	-1.050a	
VAR00007	20.1667	4.567	-.642	-.443a	
VAR00008	20.3333	5.467	-.765	-.137a	
VAR00009	21.0000	2.800	-.231	-1.232a	
VAR00010	21.3333	2.267	.000	-1.919a	

a. The value is negative due to a negative average covariance among items. This violates reliability model assumptions. You may want to check item codings.	

SCAT & CT

RELIABILITY
  /VARIABLES=VAR00001 VAR00002 VAR00003 VAR00004 VAR00005 VAR00006 VAR00007 VAR00008 VAR00009
    VAR00010 VAR00011 VAR00012 VAR00013 VAR00014 VAR00015
  /SCALE('ALL VARIABLES') ALL
  /MODEL=ALPHA
  /SUMMARY=TOTAL.


Reliability


Notes	
Output Created	04-MAR-2021 05:46:47	
Comments		
Input	Active Dataset	DataSet0	
	Filter	<none>	
	Weight	<none>	
	Split File	<none>	
	N of Rows in Working Data File	6	
	Matrix Input		
Missing Value Handling	Definition of Missing	User-defined missing values are treated as missing.	
	Cases Used	Statistics are based on all cases with valid data for all variables in the procedure.	
Syntax	RELIABILITY
  /VARIABLES=VAR00001 VAR00002 VAR00003 VAR00004 VAR00005 VAR00006 VAR00007 VAR00008 VAR00009
    VAR00010 VAR00011 VAR00012 VAR00013 VAR00014 VAR00015
  /SCALE('ALL VARIABLES') ALL
  /MODEL=ALPHA
  /SUMMARY=TOTAL.	
Resources	Processor Time	00:00:00.00	
	Elapsed Time	00:00:00.00	


[DataSet0] 


Scale: ALL VARIABLES


Case Processing Summary	
	N	%	
Cases	Valid	6	100.0	
	Excludeda	0	.0	
	Total	6	100.0	

a. Listwise deletion based on all variables in the procedure.	


Reliability Statistics	
Cronbach's Alpha	N of Items	
.878	15	


Item-Total Statistics	
	Scale Mean if Item Deleted	Scale Variance if Item Deleted	Corrected Item-Total Correlation	Cronbach's Alpha if Item Deleted	
VAR00001	20.8333	34.967	.000	.883	
VAR00002	18.8333	25.367	.977	.843	
VAR00003	18.6667	25.067	.907	.848	
VAR00004	20.8333	34.967	.000	.883	
VAR00005	18.5000	28.300	.691	.863	
VAR00006	18.6667	27.467	.879	.852	
VAR00007	20.8333	34.967	.000	.883	
VAR00008	19.3333	28.667	.625	.867	
VAR00009	18.8333	27.767	.679	.864	
VAR00010	20.8333	34.967	.000	.883	
VAR00011	18.6667	29.067	.657	.865	
VAR00012	18.1667	29.767	.876	.859	
VAR00013	20.8333	34.967	.000	.883	
VAR00014	18.5000	34.300	.000	.900	
VAR00015	19.3333	30.267	.730	.863	

NEW FILE.
DATASET NAME DataSet1 WINDOW=FRONT.
RELIABILITY
  /VARIABLES=VAR00001 VAR00002 VAR00003 VAR00004 VAR00005 VAR00006 VAR00007 VAR00008 VAR00009
  /SCALE('ALL VARIABLES') ALL
  /MODEL=ALPHA
  /SUMMARY=TOTAL.


Reliability


Notes	
Output Created	04-MAR-2021 05:47:35	
Comments		
Input	Active Dataset	DataSet1	
	Filter	<none>	
	Weight	<none>	
	Split File	<none>	
	N of Rows in Working Data File	6	
	Matrix Input		
Missing Value Handling	Definition of Missing	User-defined missing values are treated as missing.	
	Cases Used	Statistics are based on all cases with valid data for all variables in the procedure.	
Syntax	RELIABILITY
  /VARIABLES=VAR00001 VAR00002 VAR00003 VAR00004 VAR00005 VAR00006 VAR00007 VAR00008 VAR00009
  /SCALE('ALL VARIABLES') ALL
  /MODEL=ALPHA
  /SUMMARY=TOTAL.	
Resources	Processor Time	00:00:00.00	
	Elapsed Time	00:00:00.02	


[DataSet1] 


Scale: ALL VARIABLES


Case Processing Summary	
	N	%	
Cases	Valid	6	100.0	
	Excludeda	0	.0	
	Total	6	100.0	

a. Listwise deletion based on all variables in the procedure.	


Reliability Statistics	
Cronbach's Alpha	N of Items	
.676	9	


Item-Total Statistics	
	Scale Mean if Item Deleted	Scale Variance if Item Deleted	Corrected Item-Total Correlation	Cronbach's Alpha if Item Deleted	
VAR00001	21.1667	20.567	-.349	.745	
VAR00002	21.6667	11.867	.830	.520	
VAR00003	21.1667	10.967	.937	.479	
VAR00004	22.5000	18.700	-.057	.721	
VAR00005	21.1667	15.367	.645	.610	
VAR00006	21.6667	12.267	.762	.540	
VAR00007	21.6667	19.867	-.214	.771	
VAR00008	21.1667	14.567	.527	.612	
VAR00009	21.1667	16.167	.278	.665	

NEW FILE.
DATASET NAME DataSet2 WINDOW=FRONT.
RELIABILITY
  /VARIABLES=VAR00001 VAR00002 VAR00003
  /SCALE('ALL VARIABLES') ALL
  /MODEL=ALPHA
  /SUMMARY=TOTAL.


Reliability


Notes	
Output Created	04-MAR-2021 06:11:02	
Comments		
Input	Active Dataset	DataSet2	
	Filter	<none>	
	Weight	<none>	
	Split File	<none>	
	N of Rows in Working Data File	6	
	Matrix Input		
Missing Value Handling	Definition of Missing	User-defined missing values are treated as missing.	
	Cases Used	Statistics are based on all cases with valid data for all variables in the procedure.	
Syntax	RELIABILITY
  /VARIABLES=VAR00001 VAR00002 VAR00003
  /SCALE('ALL VARIABLES') ALL
  /MODEL=ALPHA
  /SUMMARY=TOTAL.	
Resources	Processor Time	00:00:00.00	
	Elapsed Time	00:00:00.03	


[DataSet2] 


Scale: ALL VARIABLES


Case Processing Summary	
	N	%	
Cases	Valid	6	100.0	
	Excludeda	0	.0	
	Total	6	100.0	

a. Listwise deletion based on all variables in the procedure.	


Reliability Statistics	
Cronbach's Alpha	N of Items	
.845	3	


Item-Total Statistics	
	Scale Mean if Item Deleted	Scale Variance if Item Deleted	Corrected Item-Total Correlation	Cronbach's Alpha if Item Deleted	
VAR00001	5.5000	3.100	.726	.774	
VAR00002	6.0000	3.200	.746	.750	
VAR00003	5.5000	3.900	.679	.821	

RELIABILITY
  /VARIABLES=VAR00001 VAR00002 VAR00003
  /SCALE('ALL VARIABLES') ALL
  /MODEL=ALPHA
  /SUMMARY=TOTAL.


Reliability


Notes	
Output Created	04-MAR-2021 06:12:01	
Comments		
Input	Active Dataset	DataSet2	
	Filter	<none>	
	Weight	<none>	
	Split File	<none>	
	N of Rows in Working Data File	6	
	Matrix Input		
Missing Value Handling	Definition of Missing	User-defined missing values are treated as missing.	
	Cases Used	Statistics are based on all cases with valid data for all variables in the procedure.	
Syntax	RELIABILITY
  /VARIABLES=VAR00001 VAR00002 VAR00003
  /SCALE('ALL VARIABLES') ALL
  /MODEL=ALPHA
  /SUMMARY=TOTAL.	
Resources	Processor Time	00:00:00.02	
	Elapsed Time	00:00:00.00	


Scale: ALL VARIABLES


Case Processing Summary	
	N	%	
Cases	Valid	6	100.0	
	Excludeda	0	.0	
	Total	6	100.0	

a. Listwise deletion based on all variables in the procedure.	


Reliability Statistics	
Cronbach's Alphaa	N of Items	
-.113	3	

a. The value is negative due to a negative average covariance among items. This violates reliability model assumptions. You may want to check item codings.	


Item-Total Statistics	
	Scale Mean if Item Deleted	Scale Variance if Item Deleted	Corrected Item-Total Correlation	Cronbach's Alpha if Item Deleted	
VAR00001	4.1667	1.367	.082	-.585a	
VAR00002	5.0000	2.400	-.158	.167	
VAR00003	4.1667	1.767	-.072	-3.997E-15a	

a. The value is negative due to a negative average covariance among items. This violates reliability model assumptions. You may want to check item codings.	

RELIABILITY
  /VARIABLES=VAR00001 VAR00002 VAR00003
  /SCALE('ALL VARIABLES') ALL
  /MODEL=ALPHA
  /SUMMARY=TOTAL.


Reliability


Notes	
Output Created	04-MAR-2021 06:12:31	
Comments		
Input	Active Dataset	DataSet2	
	Filter	<none>	
	Weight	<none>	
	Split File	<none>	
	N of Rows in Working Data File	6	
	Matrix Input		
Missing Value Handling	Definition of Missing	User-defined missing values are treated as missing.	
	Cases Used	Statistics are based on all cases with valid data for all variables in the procedure.	
Syntax	RELIABILITY
  /VARIABLES=VAR00001 VAR00002 VAR00003
  /SCALE('ALL VARIABLES') ALL
  /MODEL=ALPHA
  /SUMMARY=TOTAL.	
Resources	Processor Time	00:00:00.02	
	Elapsed Time	00:00:00.03	


Scale: ALL VARIABLES


Case Processing Summary	
	N	%	
Cases	Valid	6	100.0	
	Excludeda	0	.0	
	Total	6	100.0	

a. Listwise deletion based on all variables in the procedure.	


Reliability Statistics	
Cronbach's Alpha	N of Items	
.750	3	


Item-Total Statistics	
	Scale Mean if Item Deleted	Scale Variance if Item Deleted	Corrected Item-Total Correlation	Cronbach's Alpha if Item Deleted	
VAR00001	6.0000	2.000	.447	.800	
VAR00002	6.0000	1.600	.750	.500	
VAR00003	6.0000	1.200	.612	.667	
